# Standardizing In Vitro β-Lactam Antibiotic Allergy Testing with Synthetic IgE

**DOI:** 10.1021/acs.analchem.3c02284

**Published:** 2023-08-07

**Authors:** Pedro Quintero-Campos, Roberto Gozalbo-Rovira, Jesús Rodríguez-Díaz, Ángel Maquieira, Sergi Morais

**Affiliations:** † Instituto Interuniversitario de Investigación de Reconocimiento Molecular y Desarrollo Tecnológico (IDM), Universitat Politècnica de València-Universitat de València, 46022 Valencia, Spain; ‡ Departamento de Microbiología, Facultad de Medicina, Universidad de València, Av. Blasco Ibáñez 17, 46010 València, Spain; § Hospital Clínico Universitario de Valencia, Instituto de Investigación INCLIVA, 46010 Valencia, Spain; ∥ Unidad Mixta UPV-La Fe, Nanomedicine and Sensors, IIS La Fe, Av. de Fernando Abril Martorell, 106, 46026 València, Spain; ⊥ Departamento de Química, Universitat Politècnica de València, Camino de Vera s/n, 46022 Valencia, Spain

## Abstract

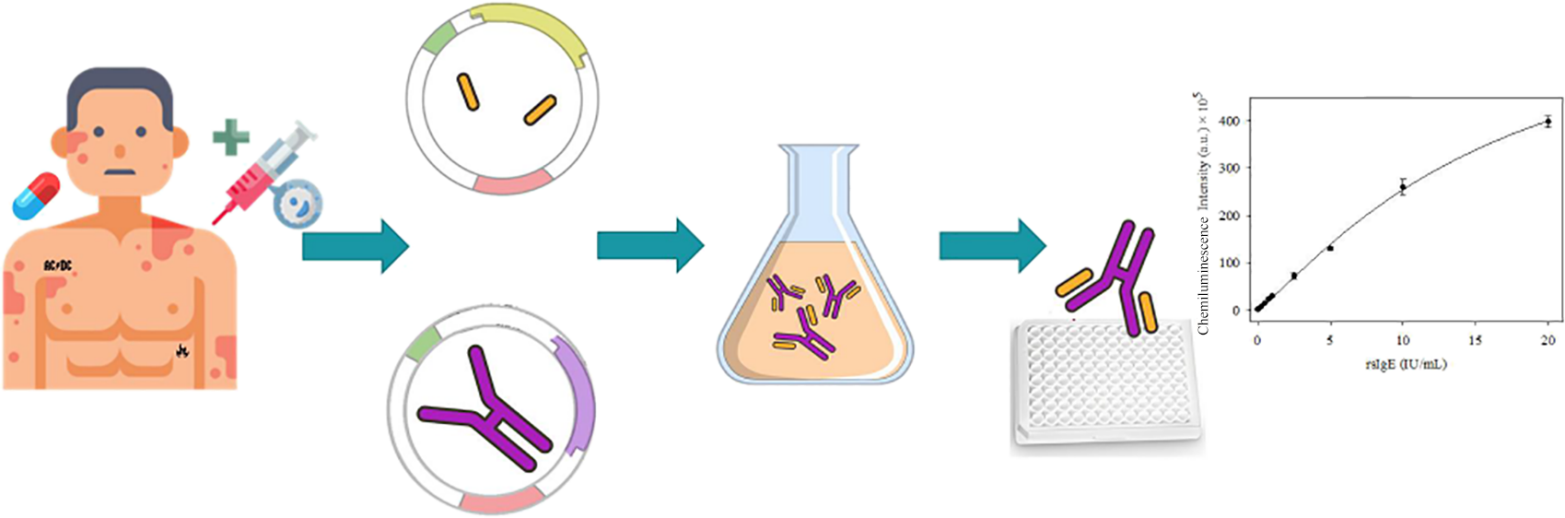

The global prevalence of β-lactam allergy poses a major challenge in administering first-line antibiotics, such as penicillins, to a significant portion of the population. The lack of β-lactam IgE antibody pools with defined selectivity hampers the standardization and validation of in vitro assays for β-lactam allergy testing. To address this limitation, this study introduces a synthetic IgE specific to β-lactam antibiotics as a valuable tool for drug allergy research and diagnostic tests. Using phage display technology, we constructed a library of human single-chain antibody fragments (scFv) to target the primary determinant of amoxicillin, a widely used β-lactam antibiotic. Subsequently, we produced a complete human synthetic IgE molecule using the highly efficient baculovirus expression vector system. This synthetic IgE molecule served as a standard in an in vitro chemiluminescence immunoassay for β-lactam antibiotic allergy testing. Our results demonstrated a detection limit of 0.05 IU/mL (0.63 pM), excellent specificity (100%), and a four-fold higher clinical sensitivity (73%) compared to the in vitro reference assay when testing a cohort of 150 serum samples. These findings have significant implications for reliable interlaboratory comparison studies, accurate labeling of allergic patients, and combating the global public health threat of antimicrobial resistance. Furthermore, by serving as a valuable trueness control material, the synthetic IgE facilitates the standardization of diagnostic tests for β-lactam allergy and demonstrates the potential of utilizing this synthetic strategy as a promising approach for generating reference materials in drug allergy research and diagnostics.

## Introduction

The oral ingestion of penicillins remains the leading cause of immune-mediated drug reactions, with approximately 10% of the general population reporting allergies to β-lactam antibiotics.^1^ However, accurate clinical and analytical assessment reveals a significant misclassification of individuals as allergic, leading to unnecessary prescription of alternative antibiotics. This mislabeling triggers socioeconomic and health problems, such as antibiotic resistance, necessitating delabeling initiatives in antibiotic stewardship programs.^2,3^

Delabeling initiatives encompass both in vivo and in vitro testing approaches. However, the routine clinical application of these methods is constrained by the time-consuming and risky nature of in vivo tests and the low sensitivity (approximately 81% false negatives) exhibited by current in vitro techniques.^4^ The first immunodiagnostic assay developed for IgE was the radioallergosorbent test (RAST), which has become outdated due to the drawbacks associated with the use of radioactive isotopes, its ineffectiveness, and its high cost.^5^ Subsequently, several alternative testing methods have been devised, focusing on the immunodetection of allergen-specific IgE (sIgE) to enhance in vitro diagnostic assays.^6^ Additionally, alternatives based on the measurement of cellular markers’ activation have emerged, such as the basophil activation test (BAT), which assesses the activation of CD63 and CD203. Although BAT offers considerable specificity, it presents complexity in its execution, thus restricting its utilization to cases where immunoassays are not feasible.^7,8^

Currently, ImmunoCAP is used as the reference method. However, discrepancies between in vitro tests have been reported.^9^ These discrepancies are attributed to variations in the presentation of the antigenic determinant on the solid phase, among other factors. Therefore, there is a need for well-defined trueness control materials and standards to accurately determine specific IgE.^10,11^ Additionally, the lack of standardized methods and consistent reference materials across manufacturers and regulatory authorities challenges method standardization and comparability.^12^

The current standard for calibrating assays, such as ImmunoCAP, for serum total IgE, is the international human serum IgE standard (coded 11/234). However, its ongoing availability requires the development of replacement preparations and further evaluation in international collaborative studies.^13^ It is important to note that blood-derived biological materials, such as the international human serum IgE standard, are subject to strict regulations due to the potential transmission of infectious diseases and emerging agents.

Validation of diagnostic methods is crucial to ensure their reliability and accuracy. This issue typically involves conducting international laboratory proficiency tests or interlaboratory comparison studies to evaluate parameters such as sensitivity, specificity, trueness, and precision, critical indicators of the assay performance. However, successful validation heavily depends on the availability of consistent reference materials.^14^

Using human control sera as reference materials for testing allergies to β-lactam antibiotics presents challenges. First, acquiring an adequate and diverse range of human control sera is difficult, limiting comprehensive validation studies. The quality of human control sera may differ, introducing additional variability into the validation process. Moreover, the high cost of obtaining and maintaining suitable human control sera poses a significant concern. Rigorous protocols and careful screening procedures are necessary for production and collection, adding to the expense.

To address these challenges, synthetic materials present several advantages, such as improved availability, reproducibility, and reduced costs over human control sera in the context of β-lactam allergies. Synthetic IgE molecules tailored explicitly for this purpose can effectively overcome the limitations associated with human control sera. Synthetic reference materials can be standardized and thoroughly characterized, enhancing comparability and consistency across diagnostic assays, manufacturers, and regulatory authorities. This standardization process has the potential to significantly improve the reliability and accuracy of allergy testing for β-lactam antibiotics.

The absence of reference materials for the measurement of β-lactam specific IgE poses a significant challenge. While purified human IgE used in quantitative and qualitative assays is monoclonal, it has inherent disadvantages such as undesired cross-reactivity and lack of selectivity.^15^ Conversely, establishing a serum bank from a diverse range of allergic patients with substantial levels of IgE antibodies is impractical due to limitations in size, reproducibility of serum pools, and the significant quantity of IgE-positive sera required for each specificity.^16^ Previous attempts to develop artificial human sera, such as chimeric adaptor molecules and specific bi-nanobodies, have involved animal immunization and lack the functional structure of IgE.^17−19^

The field of analytical chemistry has been revolutionized by antibody engineering, enabling the generation of functional and selective antibodies from single-chain variable fragment (scFv) libraries. This study would provide valuable guidance for selecting alternative antibiotics by conducting diagnostic tests with alternative β-lactams. It is essential to consider that the antigenic determinant represents a critical component in drug allergy testing, and it accounts for the discrepancies observed among these tests.^20,21^ We propose the production of a synthetic IgE molecule as a viable alternative. By constructing an immune combinatorial human scFv library and using phage display technology, we selected scFv variants with high affinity and selectivity for amoxicillin. These variants were expressed as complete human IgE molecules with the desired specificity using the Sf9 baculovirus expression system.^22^ The synthetic IgE molecule was then used as a multipoint calibrator in a chemiluminescence immunoassay, enabling the analysis of 150 human serum samples for amoxicillin allergy testing.

The production of a standardized material for determining β-lactam specific IgE holds significant potential for enhancing the accuracy and reliability of allergy testing in clinical practice. It also might have implications for reducing healthcare costs, promoting personalized medicine approaches, and improving patient outcomes. In this study, we present the production of a whole synthetic IgE molecule with selectivity for amoxicillin, a commonly prescribed β-lactam antibiotic. By utilizing this synthetic IgE molecule as a standard, we aim to improve the diagnostic accuracy of amoxicillin-specific IgE antibodies, representing a significant advancement in reliable and standardized immunoassays for β-lactam antibiotic allergy testing.

## Materials and Methods

### Chemicals and Reagents

Histone H1, penicillin G (PG), amoxicillin (AMX), aztreonam (AZT), cefaclor (CFC), imipenem (IMI), isopropyl β-d-1-thio-galactopyranoside (IPTG), polyethylene glycol 8000 (PEG), 2xTY Medium, Tween 20, DNA T4 Ligase, SfiI, MvaI, and other chemicals were obtained from Sigma-Aldrich (Madrid, Spain). Mouse monoclonal antibody anti-human IgE was purchased from Eurofins Ingenasa S.A. (Madrid, Spain). Goat anti-mouse IgG (GAM-HRP) and anti-cMyc-HRP were procured from Abcam (Cambridge, United Kingdom). The enhanced chemiluminescent substrate solution was obtained from Thermo Fisher (Madrid, Spain), and the ELISA plates were purchased from Costar Corporation (Madrid, Spain).

### Phage Display

In order to produce recombinant binders that closely resemble human IgE while circumventing the need for animal immunization, a source of lymphocytes was obtained from a willing donor. This donor exhibited elevated levels of sIgE antibodies targeting the major determinant of penicillin G and amoxicillin (the -lloyl derivatives) and was diagnosed as allergic to these drugs using the European Network of Drug Allergy (ENDA) protocol. The diagnosis was based on skin testing, in vitro assessment (Rast rating 3 by ImmunoCap), and drug provocation tests. Total RNA was extracted, retrotranscribed to cDNA using the ReverAID Reverse Transcriptase (Thermo Fisher Scientific), and DNA fragments encoding the *V*_H_ and *V*_L_ of the immunoglobulins were amplified and assembled by PCR, as previously described.^23,24^ The fragments obtained were digested using SfiI, cloned into the pComb3XSS phagemid vector,^25^ and electroporated into XL1-Blue competent cells. The cells were cultured and superinfected with KM13 helper phage to generate the library.

**Figure 1:**
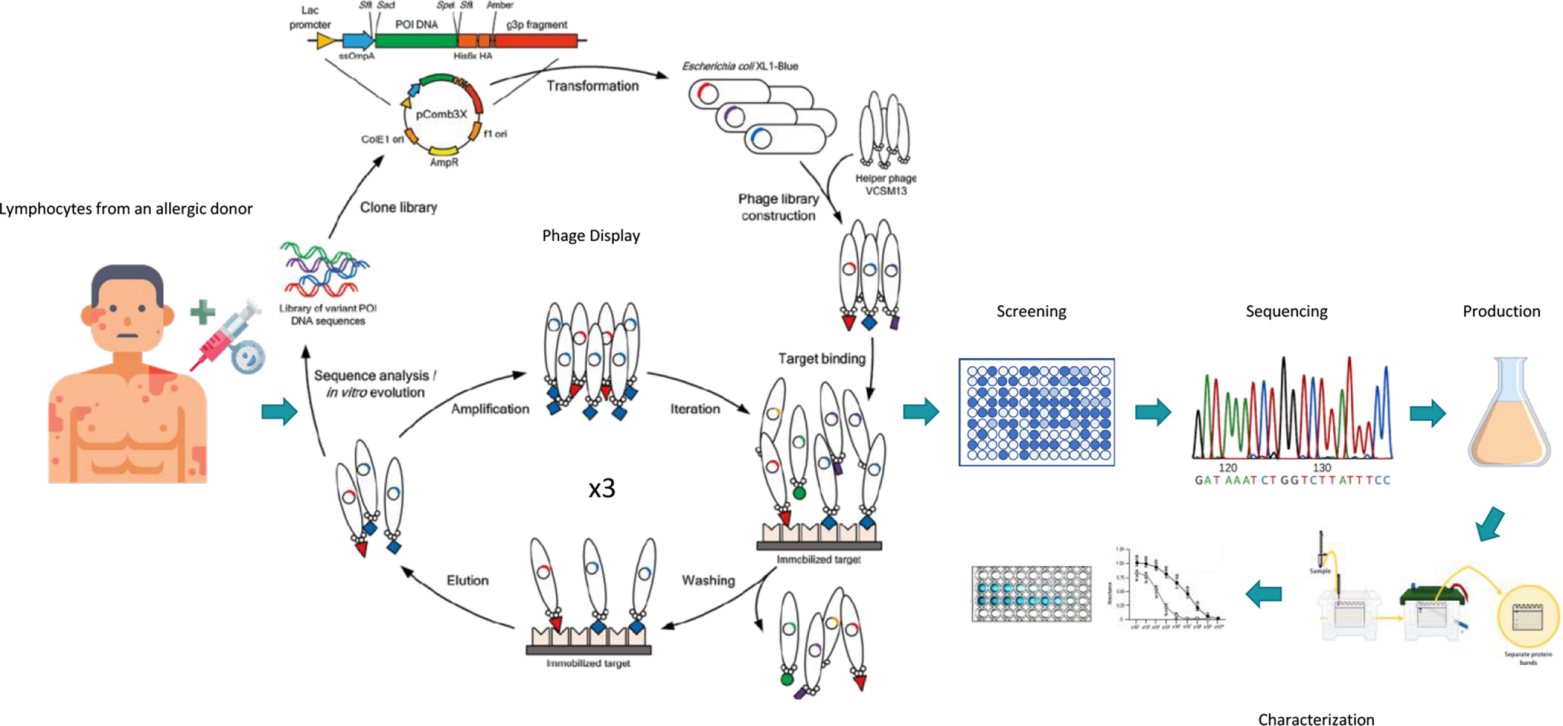
Diagram of the process of obtaining scFv by phage display. Adapted from *Phage Display of Engineered Binding Proteins* by Mark Levisson et al., 2014. Copyright 2014 by Springer Link.

High-binding plates were coated with 50 μL/well of 10 μg/mL H1-AMX conjugate in a coating buffer (50 mM sodium carbonate/bicarbonate, pH 9.6) at 4 °C for 16 h. The next day, the wells were blocked with 150 μL of TBS 3% BSA for 1 h at 37 °C, followed by washing with PBS-T 0.05%. The coated wells were incubated with phage particles of the scFv library for 2 h at 37 °C. The wells were washed 10 times, and the bound phages were eluted by incubation with 50 μL/well of trypsin at 10 mg/mL for 30 min at 37 °C. Finally, the phages were collected and used for titration and subsequent amplification in *E. coli* XL1-Blue for an additional round of panning. Individual phages obtained in the third round of panning were screened (Supporting Information) in ELISA plates coated with the H1-AMX conjugate. Bound phages were detected with an anti-M13-HRP following the protocol described in the Supporting Information. Figure 1 illustrates the process.

### Production and Characterization of scFv

The ELISA-positive clones were characterized by MvaI restriction analysis and fingerprinting.^26^ scFv genes were amplified from single *E. coli* colonies with the primers ompseq (AAGACAGCTATCGCGATTGCAG’) and dpseq (AGAAGCGTAGTCCGGAACGTC’), followed by the digestion of the PCR products with MvaI. Clones were classified according to the fingerprinting patterns and sequenced. The clones grown in 1 L of 2xTY with ampicillin (100 μg/mL) were induced with 0.5 mM IPTG for 16 h at 25 °C under shaking. The cultures were centrifuged, resuspended in PBS containing 20 mM imidazole, DNase I, and lysozyme, and sonicated. The supernatant was purified by affinity chromatography using an FPLC ÄKTA system (GE Healthcare) fitted with a His-Trap column (Thermo Fisher Scientific). The imidazole-eluted fractions were dialyzed against PBS, and their protein content was quantified by Bradford’s method. The purified scFv was analyzed by 12% sodium dodecyl sulfate-polyacrylamide gel electrophoresis (SDS-PAGE) and Coomassie brilliant blue staining method, Western blot, and ELISA, as described in the Supporting Information.

### Cloning of IgE cDNA into the Baculovirus Expression Vector

To facilitate the production of high-quality and functional synthetic IgE, we synthesized the full-length cDNA encoding the human IgE entire heavy and light chains and cloned it into the pFastBac1 vector (GeneArt Gene Synthesis, Thermo Fisher Scientific). The resulting recombinant bacmid DNA was then inserted into the genome of the baculovirus via transformation into *E. coli* DH10Bac cells, followed by plating onto LB-agar plates containing 20 μg/mL of X-gal, 40 μg/mL of IPTG, 7 μg/mL of gentamicin, 50 μg/mL of kanamycin, and 10 μg/mL of tetracycline. After selecting the white colonies, PCR was performed using M13 forward and reverse primers (CCCAGTCACGACGTTGTAAAACG and AGCGGATAACAATTTCACACAGG) to confirm successful cDNA insertion. To transfect Sf9 cells, we used five micrograms of the recombinant bacmid DNA mixed with eight microliters of FuGene HD reagent (Promega) and added it to 9 × 10^5^ exponentially growing cells. Recombinant baculoviruses were then harvested 3 days post-transfection and stored at 4 °C. The viral stocks were further amplified by infecting a suspension culture of 3 × 10^6^ Sf9 cells/mL, which were then incubated with the virus in a serum-free SFM900II medium with 1% Pluronic F-68 and appropriate antibiotics at 27 °C with orbital shaking (120 r.p.m.). The viruses were harvested at 72 h post-infection and stored at 4 °C.

### Expression, Purification, and Characterization of the Synthetic IgE

After producing the recombinant baculoviruses, we infected a culture (750 mL) of growing Sf9 cells at a density of 2.5 × 10^6^ cells/mL with the virus under shaking (120 rpm) at 27 °C. After 96 h of incubation, cell culture bottles were harvested by centrifugation at 25,000 *g* for 1 h. The supernatant was then purified by affinity chromatography using an FPLC ÄKTA system (GE Healthcare) fitted with a HiTrap protein L column (Thermo Fisher Scientific). The eluted fractions were dialyzed against PBS, and the protein content of the fractions was quantified using both spectrophotometry (Abs 280 nm 0.1% = 1.9) and the Bradford method. The purified IgE was then analyzed by Western blot and ELISA, as described in the Supporting Information.

### Analysis of Serum Samples

To evaluate the efficacy of our synthetic IgE, we analyzed a cohort of 150 human serum samples collected from consenting donors according to standardized protocols by Hospital Universitari i Politècnic La Fe (Valencia, Spain). The participants were enrolled after giving written informed consent according to protocols approved by the ethics review board at Hospital Universitari i Politècnic La Fe (registry no. COBIOPHAD), and all procedures were performed following the Helsinki Declaration of 1975, as revised in 2008. To quantify the concentration of specific IgE in the collected serum samples, we developed a dose–response chemiluminescence immunoassay (CLIA)^27^ using our synthetic IgE (St-IgE) as a multipoint standard. White-bottomed polystyrene ELISA plates were coated with H1-AMX (3.0 mg/mL) in coating buffer (25 μL/well) and incubated overnight at 4 °C. The next day, the plates were washed four times with PBS-T, and 25 μL/well of sera or st-IgE was added to each well, followed by incubation for 30 min at room temperature. After the wells were washed, 25 μL of anti-human IgE monoclonal antibody solution (1/2000 dilution) was added. After 15 min, the plate was washed, and preabsorbed goat anti-rat IgG solution (1/500) was added to each well and incubated under the same conditions. Finally, after the plate was washed, peroxidase activity was measured by adding 25 μL of the enhanced chemiluminescent substrate solution previously diluted 1/10 in PBS. Luminescence signals were read at 450 nm using a multimode plate reader.

The homologous calibration strategy allowed us to accurately measure the concentration of IgE in each sample and evaluate the method’s performance.

## Results

### Production and Characterization of scFv

**Figure 2:**
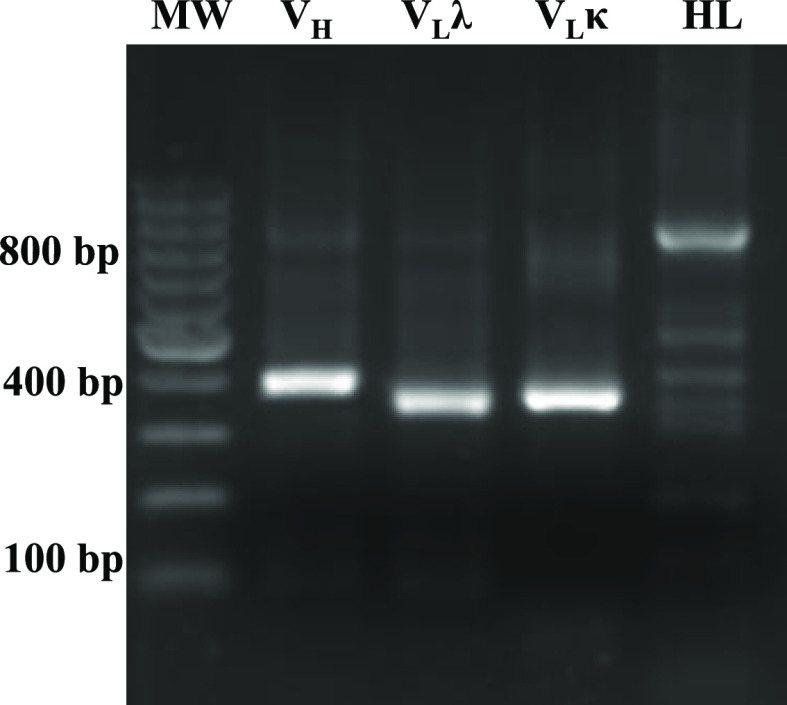
Amplification and assembly of scFv. PCR products for amplification of the *V*_H_ and *V*_L_ (*V*_L_λ and *V*_L_κ) and their assembly into scFv (*H*_L_).

β-Lactam antibiotics can elicit complex patterns of drug allergy. However, despite the heterogeneous reactivity profiles found in the allergic population, the primary determinant responsible for triggering the allergic event in most cases of hypersensitivity to these antibiotics is the major determinant, as confirmed in the clinical analysis of the allergic patient from whom DNA and subsequent st-IgE were obtained. The generation of recombinant scFv monoclonal antibodies using cDNA templates derived from human lymphocytes was performed through phage display technology. PCR amplification of the variable light-chain (*V*_L_) and variable heavy-chain (*V*_H_) domains resulted in fragments of approximately 350 and 400 bp, respectively. Figure 2 depicts the PCR products obtained for the amplification of VH, variable light chain lambda (*V*_Lλ_), and variable light chain kappa (*V*_Lκ_). These products were assembled to form the single-chain variable fragment (scFv) construct, designated as *H*_L_. The two domains were connected using a long linker (GGSSRSSSSGGGGSGGGG), generating combinatorial scFv repertories with an approximate molecular size of 800 bp. These scFv coding sequences were then cloned into XL1-Blue, leading to the construction of an scFv library.

Phage antibody selection against the H1-AMX conjugate was carried out through three rounds of panning, resulting in a substantial enrichment of phage-forming units. Specifically, eluted phages increased from 2 × 10^4^ to 3.6 × 10^6^ in the third round, indicating a 180-fold enrichment. From the last round of panning, 96 phage clones were randomly selected for further testing. Through indirect ELISA, it was found that 9.4% (9/96) (Figure S1C) of these phage clones exhibited specific binding to H1-AMX, indicating their potential as targeted antibodies.

**Figure 3:**
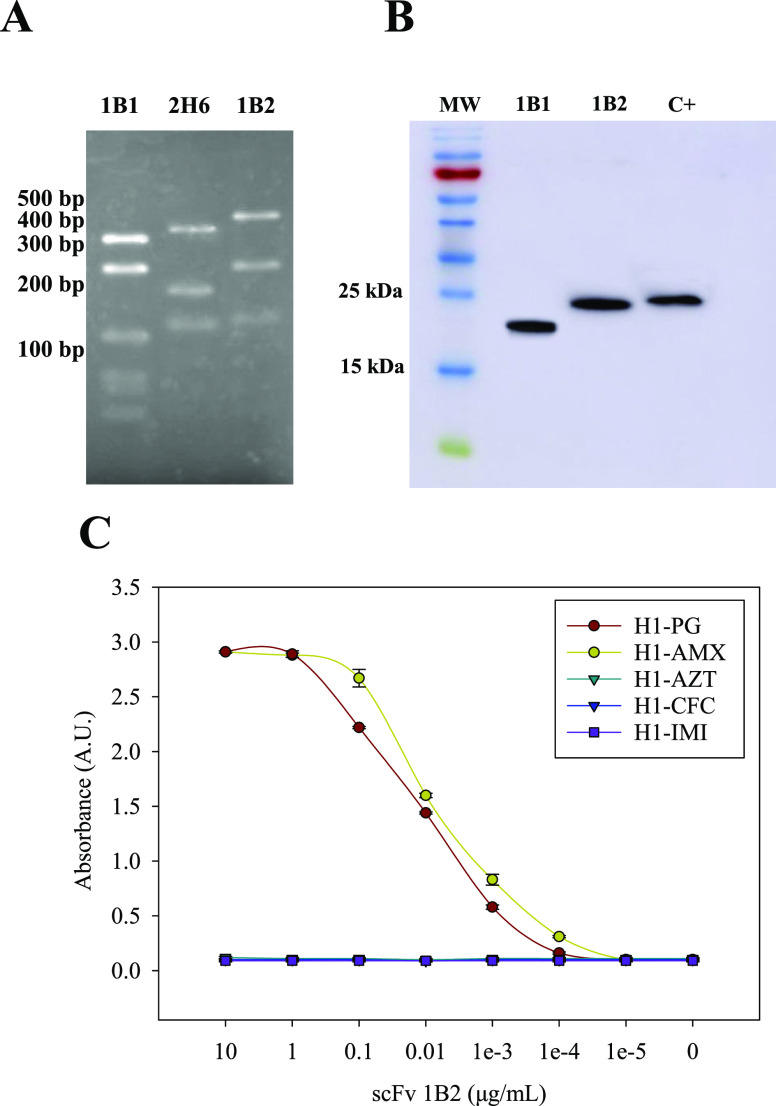
Characterization of scFv. (A) *MvaI* fingerprinting analysis was performed on the scFv genes, and the results were resolved using a 3% agarose gel. (B) Western blot analysis of purified scFv. (C) Reactivity of the scFv was tested against H1 conjugates of PG, AMX, AZT, CFC, and IMI, with the last three serving as negative control antigens.

Following the selection of phage clones, the scFv genes from the chosen clones were amplified by PCR and subjected to MvaI restriction analysis. Analysis of the resulting bands (Figure 3A) revealed three distinct patterns. Figure 3A presents the MvaI fingerprinting analysis of the scFv genes, visualized on a 3% agarose gel. This analysis revealed distinct banding patterns, confirming the successful generation of different scFv clones. Sequence analysis confirmed that clones 1B1 and 1B2 possessed both the *V*_H_ and *V*_L_ regions, while clone 2H6 only had the *V*_L_ region. The clones with correct sequences were subsequently produced and purified according to the methods described above. The Western blot analysis shown in Figure 3B demonstrates the purification of the scFv clones. Clone 1B2 displayed a clear and intense band at approximately 20 kDa, corresponding to the expected molecular weight of the scFv, whereas clone 1B1 exhibited a slightly smaller size than anticipated.

Moreover, the binding affinities of each clone toward the H1-AMX, H1-PG, H1-AZT, H1-CFC, and H1-IMI conjugates were assessed by ELISA. Clone 1B2 (Figure 3C) demonstrated specific recognition toward H1-AMX and H1-PG while displaying no binding to H1-AZT, H1-CFC, and H1-IMI (negative control determinants), thus indicating its selectivity. Conversely, clone 1B1 did not display specific recognition for the H1-AMX or H1-PG conjugates (Figure S2). These findings validate the remarkable selectivity of clone 1B2 as it exhibits reactivity identical to that of the donor.

An advantage of our approach lies in the construction of an immune scFv library, providing virtually unlimited access to allergen-specific antibodies necessary for synthetic sera production. Although the IgE repertoire in human sera may recognize specific and potentially restricted epitopes, the significantly higher clonality of the scFv repertoire is likely sufficient to encompass all IgE epitopes.

### Expression, Purification, and Characterization of St-IgE

**Figure 4:**
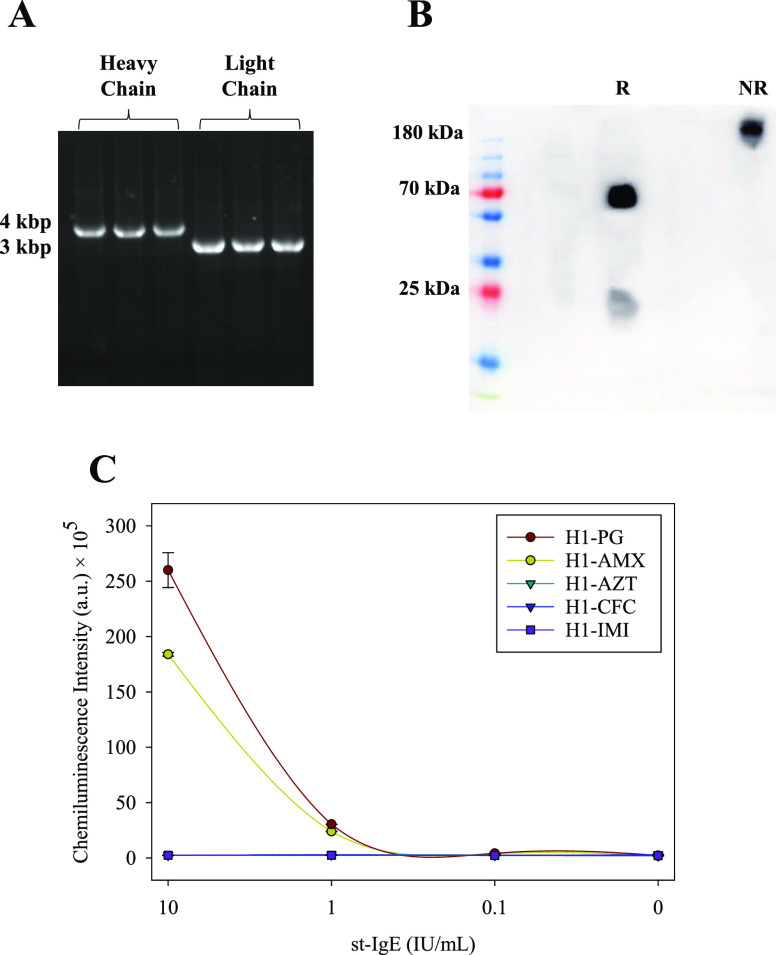
Expression and characterization of St-IgE. (A) PCR products that confirm the pFast-Bac1 plasmids containing the light and heavy chain. (B) Western blot analysis after SDS-PAGE of purified St-IgE. The St-IgE was analyzed in reducing (R) and non-reducing (NR) conditions. (C) Analysis of St-IgE antibodies’ binding specificity by ELISA. H1-AZT, H1-CFC, and H1-AZT serve as negative control determinants.

The pFast-Bac1 plasmids containing the light- and heavy-chain sequences were transformed into DH10-Bac cells and confirmed by PCR analysis. Figure 4A confirms the presence of the pFast-Bac1 plasmids containing the light and heavy chains, as demonstrated by the PCR products obtained. The products’ sizes correspond to the plasmids’ expected sizes with the inserted chains. As illustrated, the PCR product obtained from colonies transformed with the light-chain plasmid had a size of 3.0 kbp, while the heavy-chain plasmid yielded a product of 4.0 kbp. Successful transformation and transposition were confirmed as the product sizes matched the size of the pFAST-Bac vector (2.3 kbp) plus the insert size.

Recombinant baculoviruses expressing the heavy and light chains of St-IgE were generated and used to infect Sf9 cells. The purified St-IgE was quantified using Nanodrop and analyzed through SDS-PAGE and Western blotting. Under the experimental conditions, one culture liter produced one soluble synthetic IgE milligram. The Western blot analysis in Figure 4B shows the purified St-IgE after SDS-PAGE under reducing and non-reducing conditions. The 20 and 70 kDa bands correspond to the light and heavy chains. Under non-reducing conditions, St-IgE appears as a complete IgE molecule with a size of 190 kDa, consistent with the expected mass of a complete IgE molecule. The full sequence of the IgE is disclosed in the Supporting Information.

Figure 4C depicts the selectivity results of St-IgE. St-IgE specifically recognizes the H1-AMX conjugate, with lower recognition of the H1-PG conjugate. No binding is observed with the H1-AZT, H1-CFC, and H1-IMI conjugates (negative controls), indicating the preservation of the scFv’s biochemical properties and the donor’s reactivity. In this case, the β-lactam ring is cleaved, and the resulting carboxyl group is linked to an amine in the carrier protein, producing the major determinant (the -lloyl derivative) that has been postulated to be the real precursor for this type of allergy.^28^ The composition of the carrier molecule and the extent of antibiotic exposure influence the binding of IgE. Two major determinants were utilized to investigate these factors, each employing distinct carrier molecules: human serum albumin (HSA) and a lysine-rich protein (H1). HSA is an endogenous protein involved in antigen presentation mechanisms upon covalent binding of drugs, whereas H1 contains numerous primary amines facilitating the coupling of β-lactam antibiotics. These approaches have led to highly sensitive assays.^11^ As depicted in Figure S3, both antigenic determinants (HSA-PG and H1-PG) exhibit similar behavior. However, when examining the antigenic determinants for AMX, the H1-AMX determinant demonstrates superior analytical performance compared to the HSA-AMX conjugate. This discrepancy may be attributed to the higher abundance of free amines available for amoxicillin conjugation in histone. The observed behavior of st-IgE aligns with the findings from the analysis of multiple samples collected from allergic patients.^11^

**Figure 5:**
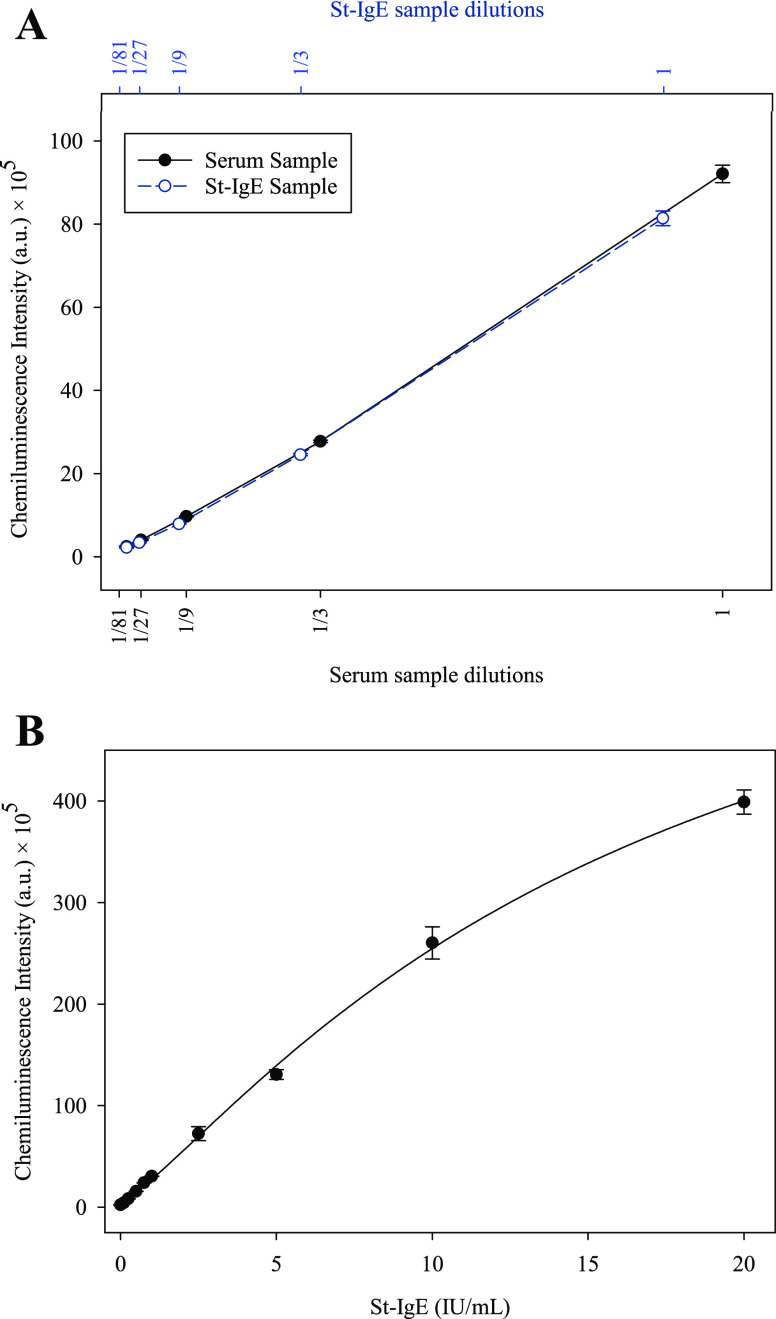
Linearity and calibration curve. (A) Dilution linearity study of a serum sample (*r*^2^ > 0.99) and St-IgE (*r*^2^ > 0.99). (B) Homologous calibration curve for sIgE to amoxicillin.

On the other hand, a linearity-of-dilution test was conducted by diluting St-IgE in sIgE-free serum to assess its performance in the CLIA assay. Additionally, the test was performed on a serum sample containing a known concentration of amoxicillin-specific IgE (3.3 IU/mL), employing three-fold serial dilutions (1-1/81). Figure 5A illustrates the linearity study of the serum sample and St-IgE, revealing a high correlation coefficient (*r*^2^ > 0.99) across a wide range of dilutions. This result demonstrates that the behavior of St-IgE and sIgE is comparable and highlights the ability of St-IgE to measure synthetic IgE concentrations accurately.

### Analysis of Serum Samples

The synthesized whole IgE molecule served as a standard to determine the concentration of specific IgE to amoxicillin in serum samples using the homologous CLIA method. Figure 5B displays the homologous calibration curve for specific IgE (sIgE) to amoxicillin, constructed using St-IgE. The curve enables the quantification of sIgE levels in patient samples. The homologous calibration curve, ranging from 0.01 to 20 IU/mL, was built by diluting St-IgE in a pooled control serum. The calibration curve demonstrated a dynamic response from 0.1 to 20 IU/mL, with a detection limit of 0.05 IU/mL (0.63 pM), surpassing the current internationally accepted cut-off for diagnosing allergy to β-lactam antibiotics.

A cohort of 150 sera collected from 75 amoxicillin-allergic patients and 75 non-allergic control subjects were analyzed in triplicate using the CLIA assay to evaluate the diagnostic performance. The obtained signals were interpolated in the homologous calibration curve. The results (Table 1) were compared with IgE measurements obtained using the current reference method (ImmunoCAP). Excellent recovery figures ranging from 81 to 120% were also obtained compared to the reference approach.

**Table 1:** Concentrations of Specific IgE to Amoxicillin Determined by CLIA and ImmunoCAP (ICAP)^a^

donor	culprit drug	sex	age	CLIA (IU/mL)	ICAP (IU/mL)	donor	culprit drug	sex	age	CLIA (IU/mL)	ICAP (IU/mL)
1	Amx.	F	63	30.30 ± 0.51	28.70	**39**	Aug.	F	82	0.40 ± 0.03	0.00
2	Aug.	M	49	0.30 ± 0.02	0.02	**40**	Amx.	F	59	<0.05	0.06
3	Aug.	F	50	0.40 ± 0.16	0.40	**41**	Aug.	M	29	<0.05	0.01
4	Amx.	F	48	<0.05	0.06	**42**	Amx.	F	32	0.20 ± 0.03	0.01
5	Aug.	F	49	1.10 ± 0.07	0.08	**43**	Aug.	M	49	0.30 ± 0.02	0.03
6	Amx.	F	55	0.65 ± 0.02	0.33	**44**	Aug.	F	38	0.55 ± 0.09	0.01
7	Aug.	M	70	0.95 ± 0.07	0.95	**45**	Aug.	F	46	0.65 ± 0.07	0.07
8	Amx.	F	38	0.75 ± 0.01	0.07	**46**	Amx.	F	58	0.45 ± 0.02	0.04
9	Amx.	F	60	<0.05	0.00	**47**	Cfr.	M	32	<0.05	0.07
10	Aug.	F	34	<0.05	0.03	**48**	Aug.	M	72	0.90 ± 0.05	0.00
11	Amx.	F	69	1.80 ± 0.02	1.65	**49**	Aug.	F	73	1.60 ± 0.08	0.00
12	Amx.	F	39	1.45 ± 0.19	0.04	**50**	Amx.	F	54	1.15 ± 0.10	0.00
13	Amx.	F	49	0.20 ± 0.01	0.05	**51**	Aug.	M	50	0.15 ± 0.01	0.01
14	Amx.	F	68	0.70 ± 0.02	0.65	**52**	Aug.	F	61	0.40 ± 0.02	0.03
15	Amx.	F	51	<0.05	0.04	**53**	Aug.	M	65	1.10 ± 0.04	0.95
16	Amx.	M	42	<0.05	0.02	**54**	Aug.	F	39	1.95 ± 0.16	0.00
17	Pen.	F	66	<0.05	0.06	**55**	Amx.	M	59	<0.05	0.05
18	Amx.	F	46	<0.05	0.03	**56**	Amx.	F	50	1.25 ± 0.15	0.09
19	Aug.	F	55	0.30 ± 0.01	0.22	**57**	Aug.	M	49	0.20 ± 0.01	0.03
20	Aug.	M	61	0.35 ± 0.01	0.05	**58**	Amx.	M	43	0.20 ± 0.01	0.05
21	Aug.	F	47	<0.05	0.00	**59**	Aug.	F	66	<0.05	0.01
22	Amx.	M	51	1.40 ± 0.01	0.87	**60**	Aug.	F	60	3.80 ± 0.15	0.09
23	Amx.	F	68	2.85 ± 0.01	0.08	**61**	Aug.	F	43	0.20 ± 0.01	0.01
24	Amx.	F	38	0.25 ± 0.01	0.01	**62**	Aug.	M	57	0.20 ± 0.02	0.08
25	Aug.	M	75	1.40 ± 0.38	0.04	**63**	Amx.	M	45	0.40 ± 0.10	0.46
26	Aug.	F	74	<0.05	0.00	**64**	Amx.	F	50	<0.05	0.02
27	Amx.	F	64	0.30 ± 0.01	0.08	**65**	Amx.	M	60	<0.05	0.03
28	Amx.	M	52	0.20 ± 0.01	ND	**66**	Aug.	M	46	0.50 ± 0.06	0.08
29	Amx.	F	57	19.50 ± 0.79	ND	**67**	Aug.	M	42	<0.05	0.03
30	Aug.	M	74	0.45 ± 0.03	0.09	**68**	Aug.	F	32	0.40 ± 0.02	0.09
31	Amx.	F	53	0.15 ± 0.01	0.01	**69**	Aug.	F	57	<0.05	0.05
32	Aug.	M	52	9.45 ± 0.05	10.20	**70**	Amx.	M	53	<0.05	0.09
33	Cfx.	F	77	1.05 ± 0.02	0.09	**71**	Amx.	M	39	0.40 ± 0.02	0.04
34	Aug.	M	71	0.30 ± 0.02	0.02	**72**	Amx.	M	39	0.80 ± 0.02	0.99
35	Aug.	F	59	<0.05	0.01	**73**	Aug.	F	39	7.05 ± 0.23	7.02
36	Aug.	F	43	6.95 ± 0.01	0.06	**74**	Aug.	F	37	0.40 ± 0.01	0.04
37	Aug.	M	54	0.20 ± 0.01	0.01	**75**	Aug.	F	49	0.25 ± 0.05	0.09
38	Aug.	M	85	0.70 ± 0.06	0.00						

a Amoxicillin: Amx.; augmentine: Aug.; cefuroxime: Cfx.; ceftriaxone: Cfr.; penicillin: Pen.; M: male; F: female; ND: not determined.

**Figure 6:**
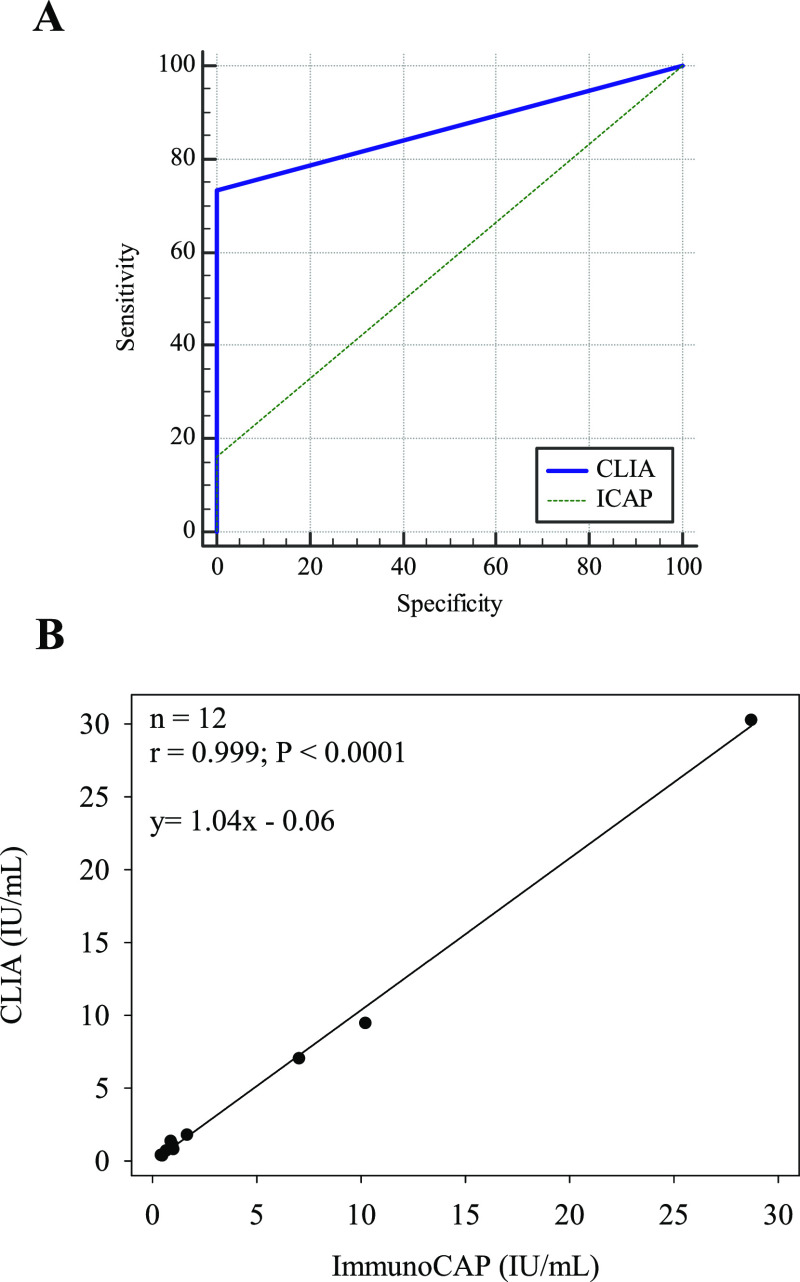
Performance analysis. (A) ROC analysis representing the area under the curve (AUC). Sensitivity and specificity of the CLIA as compared against ImmunoCAP (*n* = 150 values). (B) Scatter diagram and regression line of inter-method comparison between CLIA with homologous calibration and reference method (ImmunoCAP).

The ability of our assay to discriminate between allergic and non-allergic individuals was also assessed. Analysis of the non-allergic control samples indicated that CLIA identified all control samples as negative, with results below the limit of detection (LOD), demonstrating excellent specificity (100%). Figure 6A shows the receiver operating characteristic (ROC) analysis, representing the area under the curve (AUC) for CLIA. The AUC demonstrates the diagnostic performance of CLIA in distinguishing between allergic and non-allergic individuals. Our assay exhibited an excellent clinical sensitivity of 73%, a substantial improvement over the ImmunoCAP assay (16%), yielding a remarkable fourfold increase. The remarkable clinical sensitivity of our assay represents a substantial advancement compared to that of the ImmunoCAP assay. This significant improvement is evidenced by the successful identification of 55 out of 75 positive samples, showcasing a remarkable increase in detection capability compared to the reference ImmunoCAP assay, which only managed to detect 12 positive samples. These results underscore the enhanced performance and diagnostic capability of the developed approach. The ability to accurately identify a higher proportion of positive samples indicates the superior sensitivity of the developed assay, allowing for more reliable and accurate identification of allergy patients. This substantial increase in diagnostic capability is particularly noteworthy as it has the potential to significantly impact clinical decision-making, treatment strategies, and patient outcomes. By surpassing the limitations of the reference ImmunoCAP assay, our approach opens new avenues for more precise and effective diagnostic procedures in drug allergy, suggesting that our assay can improve early detection rates and reduce false negatives significantly. This result has implications for timely intervention, improved patient management, and better overall health outcomes.

Figure 6B presents a scatter diagram and regression line comparing the results obtained by CLIA with homologous calibration and the reference method (ImmunoCAP). The strong correlation (*r* = 0.999) also indicates the reliability and agreement between the two methods. Notably, all positive results from ImmunoCAP were also positive by CLIA.

## Conclusions

Our findings present a paradigm-shifting breakthrough in drug allergy research, specifically in the context of β-lactam antibiotic allergies. Through our study, we have achieved a remarkable feat by producing an artificially tailored IgE with high affinity and selectivity for amoxicillin. This accomplishment showcases the potential and feasibility of our approach, offering a new avenue for developing targeted and standardized immunoassays for β-lactam allergy testing.

The successful validation of our synthetic IgE sets the stage for future advancements in drug allergy, paving the way for the development of specific in vitro tests that can effectively detect and differentiate β-lactam allergies from other conditions. By standardizing these immunoassays, we can ensure consistency and comparability across different laboratories and regulatory authorities, ultimately enhancing the reliability of diagnostic testing.

Moreover, the synthetic IgE molecule can be generated to exhibit reactivity toward any β-lactam antibiotic, enabling cross-reactivity studies in individuals with penicillin allergies. In the case of drugs where the determinants formed after protein conjugation are not stable, such as clavulanic acid or cephalosporins, combinatorial chemistry would be employed to obtain stable determinants.^29^ This study would provide valuable guidance for selecting alternative antibiotics by conducting diagnostic tests with alternative β-lactams. It is essential to consider that the antigenic determinant represents a critical component in drug allergy testing, and it accounts for the discrepancies observed among these tests. Therefore, the synthetic IgE molecule could be pivotal in selecting new antigenic determinants for β-lactams or adducts and identifying new epitopes before developing novel in vitro tests.

These results have far-reaching implications for the scientific community and clinical practice. They provide evidence of the feasibility of generating tailored IgE molecules for specific drug allergies, opening up avenues for personalized medicine approaches. Moreover, the availability of standardized and specific immunoassays will improve patient care by enabling accurate diagnosis, reducing the risk of unnecessary alternative antibiotic prescriptions, and addressing the socioeconomic and health problems associated with mislabeling.

In conclusion, our study represents a groundbreaking example of producing an artificial IgE molecule tailored to amoxicillin using an immune human scFv library. The successful production and validation of this synthetic IgE demonstrate the feasibility and potential of our approach in developing specific and standardized immunoassays for β-lactam allergy testing. This advancement holds promise for advancing drug allergy research, improving clinical diagnostics, and benefiting patient outcomes.

Our findings align with national and international action plans to combat antibiotic-resistant bacteria by accurately identifying allergic patients and prescribing the appropriate antibiotics, ultimately reducing the spread of drug-resistant pathogens. Additionally, the development of synthetic IgE offers a promising pathway for standardizing improved in vitro allergy testing methods and supporting drug allergy research, thereby paving the way for more effective patient care.

## Supporting Information Available

Additional experimental details, materials, and methods, including photographs of phage display methodology, production of scFv, St-IgE characterization, CLIA protocol, and analysis of a cohort of serum samples (PDF)

## Abbreviations

AMX: amoxicillin
CFC: cefaclor
CLIA: chemiluminescence immunoassay
GAM: goat anti-mouse antibody
H1: histone H1
HRP: horseradish peroxidase
IgE: immunoglobulin E
IU: international units
LOD: limit of detection
LOQ: limit of quantification
PEG: polyethylene glycol 800
PFU: plaque-forming unit
PG: penicillin G
scFv: single-chain variable fragment antibodies
SDS-PAGE: sodium dodecyl sulfate-polyacrylamide gel electrophoresis
sIgE: specific IgE
TMB: 3,3′,5,5′- tetramethylbenzidine
*V*_H_: variable heavy chain
*V*_L_: variable light chain
