# Supplementary material for: Standardizing In Vitro β-Lactam Antibiotic Allergy Testing with Synthetic IgE
Source: Anal Chem. 2023 Aug 7;95(32):12113–21. doi: 10.1021/acs.analchem.3c02284 (PMC10859892; doi:10.1021/acs.analchem.3c02284)
Supplement: Supplementary file 1 — ac3c02284_si_001.pdf [file ac3c02284_si_001.pdf]

SUPPORTING INFORMATION

Standardizing *In Vitro* β-Lactam Antibiotic Allergy Testing with Synthetic IgE

Pedro Quintero-Campos,[1] Roberto Gozalbo-Rovira,[2,3] Jesús Rodríguez-Díaz,[2,3] Ángel Maquieira,[1,4,5] Sergi Morais*[1,4,5]

[1]Instituto Interuniversitario de Investigación de Reconocimiento Molecular y Desarrollo Tecnológico (IDM), Universitat Politècnica de València-Universitat de València, Spain. [2]Departamento de Microbiología, Facultad de Medicina, Universidad de València, Av. Blasco Ibáñez 17,46010 València, Spain. [3]Hospital Clínico Universitario de Valencia, Instituto de Investigación INCLIVA, 46010 Valencia, Spain. [4]Unidad Mixta UPV-La Fe, Nanomedicine and Sensors, IIS La Fe, Av. de Fernando Abril Martorell, 106, 46026 València, Spain. [5]Departamento de Química, Universitat Politècnica de València, Camino de Vera s/n, 46022 Valencia, Spain.

1. **Phage display methodology** S-2
2. **Characterization of scFv** S-4
3. **Characterization of St-IgE** S-5
4. **CLIA protocol** S-8
5. **Analysis of serum samples. Controls.** S-9

1. Phage display methodology

1.1. Antigenic determinants

The antigenic determinants were prepared to obtain the major determinant (Figure S1A). The corresponding amount of antibiotic was reacted with the carrier molecules (5.0 mg; molar ratio 1:1000) dissolved in 1.0 mL of 0.5 M sodium carbonate pH 11.0 overnight at room temperature. H1 was the carrier protein to prepare the BLC-derived antigens for the panning and analysis of serum samples. The antigens were purified using centrifugal filters (Amicon Ultra centrifugal filters) and PBS as buffer exchange, and the concentration was determined with the Bradford protein assay.

1.2. Panning strategy for the selection of scFv

High-binding plates were coated with 50 µL/well of 10 µg/mL H1-AMX, and H1-PG conjugates in a coating buffer (50 mM sodium carbonate/bicarbonate, pH 9.6) at 4 °C for 16 h. The next day, the wells were blocked with 150 µL TBS 3% BSA for 1 h at 37 °C, followed by washing with PBS-T 0.05%. The coated wells were incubated with phage particles of the scFv library for 2 h at 37 °C. The wells were washed ten times, and the bound phages were eluted by incubation with 50 µL/well of trypsin at 10 mg/mL for 30 min at 37 °C. Finally, the phages were collected and used for titration and subsequent amplification in *E. coli* XL1-Blue for an additional round of panning. A total of three rounds were performed.

Figure S1B shows the phage titration result after each panning round. As can be seen, there is an increase in phage. In the first round, the titration result is $2 \cdot 10^4$ CFU/mL. After the second round of panning, it increases to $1.4 \cdot 10^5$ CFU/mL; finally, in the third round, it reaches $2.4 \cdot 10^6$ CFU/mL.

1.3. Screening of positive clones by ELISA

After completing the panning rounds, the eluted phages were used to infect *E. coli* XL1-Blue and grown on LB/agar plates. The isolated bacterial colonies were then grown in 100 µL of 2xTY medium containing 100 µg/mL ampicillin and 1% glucose in 96 cell-well plates at 37 °C overnight.

The following day, in 96 cell-well plates, 200 µL of 2xTY medium supplemented with 100 µg/mL ampicillin and 1% glucose were inoculated with 2 µL of single cultures and incubated with shaking at 37 °C for 2 h. After that, 109 KM13 helper phage was added and incubated for 1 h. The cultures were then harvested, and the pellet was resuspended in 200 µL

of 2xTY medium containing 100 μg/mL ampicillin and 50% kanamycin. The resuspended cultures were grown by shaking overnight at 30°C.

Furthermore, high-binding plates were coated with 100 μL/well of 10 μg/mL H1-AMX and H1-PG conjugates in a coating buffer and incubated at 4 °C for 16 h. The next day, the wells were blocked with 200 μL of MPBS 2% for 2 h at room temperature, followed by washings with PBS. Next, 50 μL of each supernatant obtained after centrifugation of the cultures was added to the wells and incubated for 1 h at room temperature. Then, the wells were washed with PBS-T, and HRP-anti-M13 (1:5,000) dilution was added and incubated under the same conditions. Finally, after washing, positive clones were detected by measuring the enzymatic activity by adding 100 μL/well of HRP substrate, which consisted of a solution containing 1.0 mL of a 4.0 mg/mL solution of OPD, 60 μL of 3% H_2O_2 in water, in a total volume of 10 mL of 0.1 M acetate buffer (pH 5.5). The enzymatic reaction was stopped after 15 min by adding 50 μL of 1M H_2SO_4, and the absorbance was read at 450 nm using a multimode plate reader.

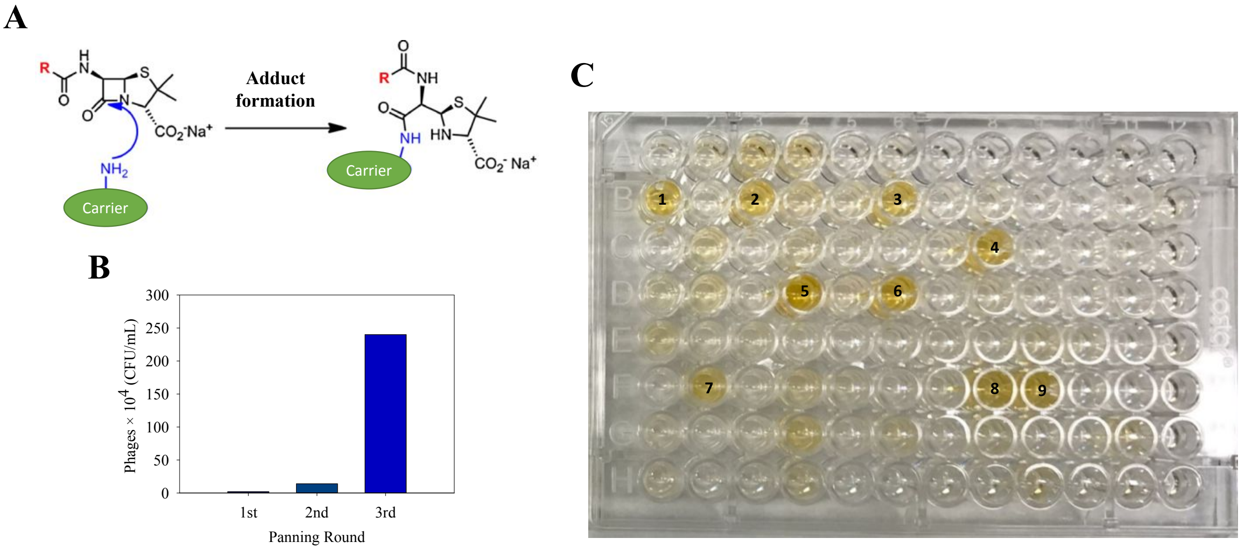

Figure S1. (A) Scheme of drug-protein adduct formation. **(B)** Result of the phage titration after each round of panning. **(C)** Image of the ELISA plate resulting from the screening performed. 9/96 of the phage clones inhibited specific binding to H1-AMX.

2. Characterization of scFv

2.1. Western Blot

The scFvs were analyzed by SDS-page using 12% gels and transferred onto nitrocellulose using a semidry system (Biorad) for 13 min at 25 mA. The scFv was detected by an anti-His monoclonal antibody (1:1,000). HRP-labeled anti-mouse antibody was used as the secondary antibody (1:5,000), and the antigen-antibody complexes were visualized by chemiluminescence with ECL (GE Healthcare).

2.2. ELISA assay to evaluate the functionality

An ELISA plate was coated for 16 h at 4 °C with 5.0 µg/mL of the H1-PG, H1-AMX, and H1-CFC conjugate in a coating buffer. After blocking with PBS-BSA 1% for 30 minutes at 37 °C, the plates were washed four times with PBS-T. Next, 100 µL/well of scFv dilutions were dispensed in PBS-BSA 0.1% and incubated for 1 hour at RT with shaking. Subsequently, after washing the plate, 100 µL/well of HRP-c-myc dilution (1:5,000) was dispensed and incubated under the same conditions. Finally, after washing the wells, the result was detected by adding HRP substrate solution.

The results in Figure S2 illustrate the reactivity of the scFv clones against H1 conjugates, including PG, AMX, and CFC as the negative control antigen. Clone 1B1 showed no specific recognition for either H1-AMX or H1-PG conjugates.

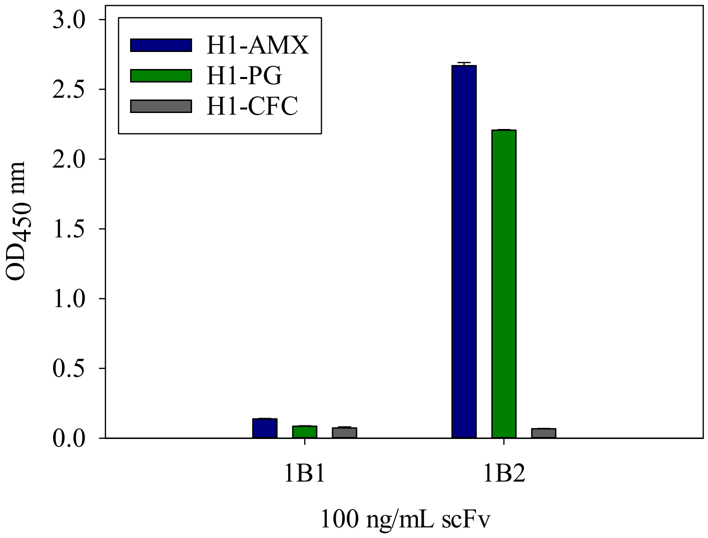

Figure S2. Reactivity of scFv against H1 conjugates to PG, AMX, and CFC, the latter as the negative control antigen.

3. Characterization of St-IgE

3.1. The amino acid sequence of the St-IgE

The complete amino acid sequence of the light and heavy chains that comprise st-IgE is the following:

Light chain

MRAWIFFLLC	LAGRALAAEL	QMTQSPSSLS	ASVGDRVTIT	CRASQSISSY
LNWYQQKPGK	APKLLIYAAS	SLQSGVPSRF	SGSGSGTDFT	LTISSLQPED
FATYYCQQSY	STPFGGGTKV	EIKRTVAAPS	VFILPPSDEQ	LKSGTASVVC
LLNNFYPREA	KVQWKVDNAL	QSGNSQESVT	EQDSKDSTYS	LSSTLTLSKA
DYEKHKVYAC	EVTHQGLSLP	VTRSFNRGEC*		

Heavy chain

MRAWIFFLLC	MRAWIFFLLC	LAGRALAAMA	LEVQLVQSGG	GLVRPGGSLR
LSCVASGFTF	SNCGMHWVRQ	SNCGMHWVRQ	APGKGLEYVS	GISSDGGSTY
YADSVKGRFT	IFRDNSKSTL	YLQVGSLRAE	DMAVYYCAKG	ESSRSAYNLD
YWGQGTLVTV	SASTQSPSVF	PLTRCCKNIP	SNATSVTLGC	LATGYFPEPV
MVTWDTGSLN	GTTMTLPATT	LTLSGHYATI	SLLTVSGAWA	KQMFTCRVAH
TPSSTDWVDN	KTFSVCSRDF	TPPTVKILQS	SCDGGGHFPP	TIQLLCLVSG
YTPGTINITW	LEDGQVMDVD	LSTASTTQEG	ELASTQSELT	LSQKHWLSDR
TYTCQVTYQG	HTFEDSTKKC	ADSNPRGVSA	YLSRPSPFDL	FIRKSPTITC
LVVDLAPSKG	TVNLTWSRAS	GKPVNHSTRK	EEKQRNGTLT	VTSTLPVGTR
DWIEGETYQC	RVTHPHLPRA	LMRSTTKTSG	PRAAPEVYAF	ATPEWPGSRD
KRTLACLIQN	FMPEDISVQW	LHNEVQLPDA	RHSTTQPRKT	KGSGFFVFSR
LEVTRAEWEQ	KDEFICRAVH	EAASPSQTVQ	RAVSVNPGK*	

3.2. Western Blot

The St-IgE was analyzed by SDS-page using 12% gels and transferred onto nitrocellulose using a semidry system for 13 min at 25 mA. St-IgE was detected by an anti-human IgE polyclonal antibody (1:1,000). An HRP-labeled anti-goat antibody was used as the secondary antibody (1:5,000), and the antigen-antibody complexes were visualized by chemiluminescence with ECL (GE Healthcare).

3.3. ELISA assay protocol to evaluate the functionality

An ELISA plate was coated for 16 h at 4 °C with 3.0 µg/mL of the H1-PG, H1-AMX, and H1-CFC conjugate in a coating buffer. The following day, the plates were washed four times with PBS-T. Next, 25 µL/well of St-IgE diluted in IgE-free human serum was dispensed and incubated for 30 min at room temperature with shaking. Subsequently, after washing the plate, 25 µL/well of monoclonal anti-human IgE solution (0.5 µg/mL) was added to each well and incubated for 15 min at room temperature. Afterward, the ELISA plate was washed, and 25 µl of goat anti-mouse IgG solution (2.0 µg/mL) was added to each well and incubated in the same conditions. Finally, the plate was washed as before, and the peroxidase activity was measured by adding 25 µL of the chemiluminescent substrate solution previously diluted 1/10 in PBS. The luminescent signals were read at 450 nm using a multimode plate reader.

3.4. Analysis of st-IgE reactivity.

The β-lactam antibiotics can give rise to complex patterns of drug allergy. However, although the reactivity profiles found in the allergic population are heterogeneous, in most cases of hypersensitivity to these antibiotics, the majority determinant is the culprit in triggering the allergic event, as occurs in the patient from whom DNA is obtained from which st-IgE is finally obtained (this was determined in the clinical analysis of the allergic patient).

In this type of determinant, the beta-lactam ring is open and linked to an amine in the carrier protein through the carboxyl group. Thus, the carrier molecule's composition and the antibiotic's exposure play a role in IgE binding. Two major determinants were used with different carrier molecules: human serum albumin (HSA) and a lysine-rich protein (H1) to study these two variants. HSA is an endogenous protein involved in antigen presentation when a drug covalently binds to it, and H1 contains dozens of primary amines that allow coupling of β-lactam antibiotics with high throughput and have resulted in very sensitive assays[1]. As seen in Figure S3, while for PG, both antigenic determinants (HSA-PG and H1-PG) show the same behavior, when AMX antigenic determinants are used, the H1-AMX determinant shows better analytical performance than the HSA-AMX conjugate. This issue may be because histone presents more free amines for amoxicillin conjugation. The behavior of st-IgE is the same as that observed in the analysis of various samples from allergic patients.[1]

Likewise, the selectivity of st-IgE for different β-lactam antibiotic conjugates (H1-Aztreonam, H1-Cefaclor, H1-Imipenem) was analyzed, and the result (Figure S3) was negative for all of them, confirming the excellent selectivity of the recombinant immunoglobulin.

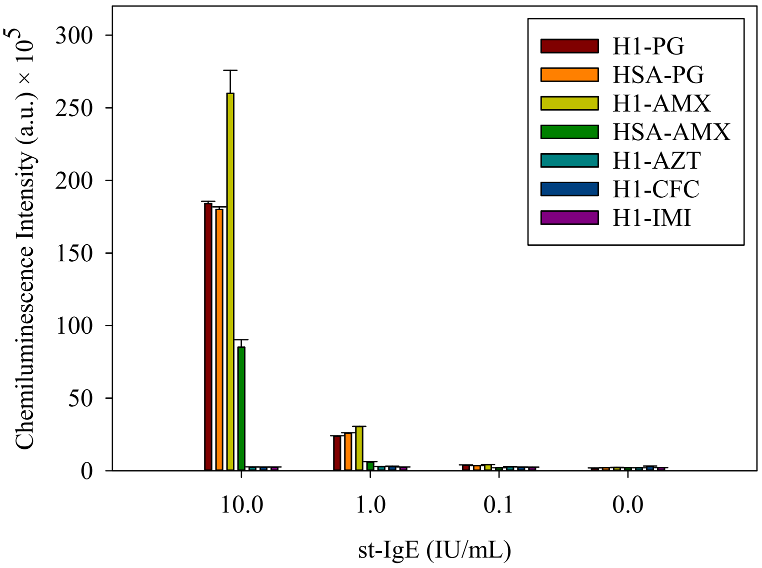

Figure S3. Analysis of st-IgE binding selectivity by CLIA. H1-AZT, H1-CFC, and H1-IMI were used as the negative control antigens.

3.5. Calibration curve for specific IgE to penicillin G.

The reactivity of st-IgE against H1-PG results in the titration curve shown in Figure S4.

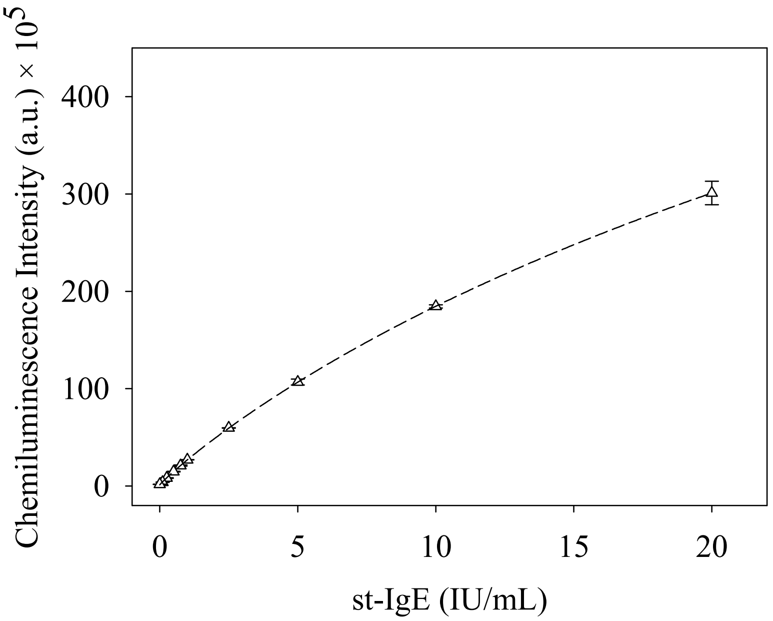

Figure S4. Titration curve of st-IgE against Penicillin G.

4. CLIA protocol

A white flat-bottomed polystyrene ELISA plate was coated with H1-AMX conjugate solutions (3.0 μg/mL) in the coating buffer (25 μL/well) to determine specific IgE to amoxicillin. The coated plates were then sealed and incubated overnight at 4 °C. On the following day, the ELISA plate was washed four times with PBS-T, and 25 μL of serum sample was added to each well, followed by incubation for 30 min at room temperature. St-IgE produced was diluted in IgE-free human serum to create a calibration curve. All serum samples were analyzed in triplicate. After washing the plate, 25 μL/well of monoclonal anti-human IgE solution (0.5 μg/mL) was added to each well and incubated for 15 min at room temperature. Subsequently, the ELISA plate was washed, and 25 μL of HRP-labeled goat anti-mouse IgG solution (2.0 μg/mL) was added to each well and incubated under the same conditions. Finally, the plate was rewashed, and the peroxidase activity was measured by adding 25 μL of the chemiluminescent substrate solution, previously diluted 1/10 (v/v) in PBS. The luminescent signals were detected at 450 nm using a multimode plate reader.

5. Analysis of serum samples. Controls.

Table S1. Concentrations of specific IgE to amoxicillin determined by CLIA and ImmunoCAP (ICAP).

Control subject	Sex	Age	CLIA (IU/mL)	ICAP (IU/mL)		Control subject	Sex	Age	CLIA (IU/mL)	ICAP (IU/mL)
**1**	F	30	< 0.05	NR		**39**	F	51	< 0.05	NR
**2**	F	53	< 0.05	NR		**40**	F	70	< 0.05	NR
**3**	M	59	< 0.05	NR		**41**	M	21	< 0.05	0.01
**4**	F	59	< 0.05	NR		**42**	F	32	< 0.05	0.01
**5**	M	66	< 0.05	NR		**43**	M	55	< 0.05	0.01
**6**	F	25	< 0.05	NR		**44**	F	35	< 0.05	0.01
**7**	M	65	< 0.05	NR		**45**	F	79	< 0.05	0.01
**8**	F	74	< 0.05	NR		**46**	F	71	< 0.05	0.06
**9**	F	54	< 0.05	NR		**47**	F	64	< 0.05	0.01
**10**	F	48	< 0.05	NR		**48**	F	60	< 0.05	0.01
**11**	F	47	< 0.05	NR		**49**	M	82	< 0.05	0.03
**12**	F	38	< 0.05	NR		**50**	F	56	< 0.05	0.00
**13**	F	40	< 0.05	NR		**51**	M	62	< 0.05	0.05
**14**	F	73	< 0.05	NR		**52**	M	63	< 0.05	0.01
**15**	F	50	< 0.05	NR		**53**	M	76	< 0.05	0.07
**16**	F	47	< 0.05	NR		**54**	M	56	< 0.05	0.03
**17**	M	72	< 0.05	NR		**55**	M	46	< 0.05	0.06
**18**	F	34	< 0.05	NR		**56**	F	69	< 0.05	0.01
**19**	F	57	< 0.05	NR		**57**	F	35	< 0.05	0.00
**20**	F	54	< 0.05	NR		**58**	F	42	< 0.05	0.00
**21**	M	46	< 0.05	NR		**59**	M	31	< 0.05	0.02
**22**	F	23	< 0.05	NR		**60**	F	61	< 0.05	0.00
**23**	F	71	< 0.05	NR		**61**	F	71	< 0.05	0.02
**24**	F	67	< 0.05	NR		**62**	M	63	< 0.05	0.00
**25**	F	67	< 0.05	NR		**63**	F	66	< 0.05	0.01
**26**	F	25	< 0.05	NR		**64**	F	49	< 0.05	0.07
**27**	F	26	< 0.05	NR		**65**	F	50	< 0.05	0.02
**28**	F	73	< 0.05	NR		**66**	F	22	< 0.05	0.00
**29**	F	36	< 0.05	NR		**67**	M	68	< 0.05	0.03
**30**	F	49	< 0.05	NR		**68**	F	58	< 0.05	0.01
**31**	F	26	< 0.05	NR		**69**	F	45	< 0.05	0.02
**32**	F	26	< 0.05	NR		**70**	F	72	< 0.05	0.00
**33**	M	63	< 0.05	NR		**71**	F	49	< 0.05	0.02
**34**	F	56	< 0.05	NR		**72**	F	55	< 0.05	0.02
**35**	F	63	< 0.05	NR		**73**	F	49	< 0.05	0.01
**36**	F	50	< 0.05	NR		**74**	F	50	< 0.05	0.02
**37**	M	39	< 0.05	NR		**75**	F	61	< 0.05	0.01
**38**	F	68	< 0.05	NR						

M: male. F: Female. NR: Not Realized (No evidence of allergy in the clinical history)
